# A New Look at an Immortal DNA Hypothesis for Stem Cell Self-Renewal

**DOI:** 10.4172/2157-7633.1000e105

**Published:** 2012-04

**Authors:** Jesse L. Mull, Atsushi Asakura

**Affiliations:** Stem Cell Institute, Paul and Sheila Wellstone Muscular Dystrophy Center, and Department of Neurology, University of Minnesota Medical School, Minneapolis, Minnesota, USA

Thirty years ago Cairns and Potten first noted that intestinal epithelial cells fail to produce carcinomas at a rate proportionate to the number of divisions they undergo throughout their lifetimes [1,2]. This observation led to the formation of the immortal DNA strand hypothesis: the suggestion that somatic stem cells segregate their DNA asymmetrically, retaining an “immortal” DNA template while passing on newly formed chromatids to daughter cells. A distinct, yet related, idea posits that somatic stem cells divide infrequently, maintaining a state of relative quiescence. In tandem, mitotic quiescence and asymmetric DNA segregation are thought to spare stem cells from accumulating mutations as they divide to replenish their respective tissues [3].

An extension of this hypothesis holds that somatic stem cells retain thymidine analog labels due to slow division rates or asymmetric segregation of DNA [4]. Preliminary experiments involving [H^3^]-thymidine incorporation into the DNA of developing mice showed that label-retaining cells persist within intestinal crypts and hair follicles over an extended period of time [3,5]. Following these initial studies, many researchers have employed similar strategies in an attempt to identify label-retaining cells and track asymmetric DNA distribution in a variety of tissues [6–20].

Nevertheless, the immortal DNA strand hypothesis remains a subject of contention, proving difficult to confirm or refute. This controversy is due in part to ill-defined or heterogeneous stem cell populations and the complexity associated with the use of thymidine analog labeling to track DNA segregation in metazoans. More importantly, few studies to date have established the fate of both parent and daughter cells following asymmetric division.

Thymidine analog labeling relies on the assumption that immortal DNA templates are labeled during symmetric stem cell divisions and that subsequent divisions, in which the labeled template is retained, will be asymmetric. However, stem cells may undergo symmetric, asymmetric, or a combination of symmetric and asymmetric divisions during development and adulthood. Therefore, the time frame of label delivery is critical for proper experimental design, and thymidine analog labeling requires precise knowledge of the time during development or regeneration when stem cells switch from symmetric to asymmetric divisions. Untimely administration or withdrawal of a label will result in its failed incorporation into template DNA strands or premature dilution, confounding experimental analysis.

To date, both growth and injury models have been employed in an attempt to overcome these obstacles. Growth models rely on the administration of a label at some time point during development, when the majority of stem cells are thought to be undergoing symmetric divisions. In contrast, injury models utilize physical or chemical means to induce a proliferative state in which symmetric stem cell divisions can occur. Recently, it has been suggested that the toxicity associated with 5-bromo-2′-deoxyuridine (BrdU) and other thymidine analogs is sufficient to induce the degree of injury required to stimulate symmetric stem cell divisions and label incorporation [7]. However, it is important to note that this toxicity may also lead to inefficient or incomplete labeling of the stem cell population due to perturbation of the cell cycle. This highlights the need for appropriate controls to ensure proliferation is not disrupted during thymidine analog labeling, as stem cells must continue to divide after successful administration of a label.

Proliferative markers like Ki67, phospho-histone H3, and proliferating cell nuclear antigen (PCNA) have been used in conjunction with stem cell markers to ensure continuous cell division, although one caveat to this strategy is the lack of appropriate markers for many stem cell populations [14]. With the recent availability of novel thymidine analogs in addition to BrdU, such as EdU, IdU and CldU, dual labeling of both the immortal DNA strand and newly synthesized DNA has also been used to establish continued cell proliferation [8–10, 12].

Recent evidence also indicates that chromosome number, as well as the number of divisions prior to analysis, can influence experimental outcomes [11]. Successive rounds of division dilute thymidine analog labels over time, leading to an increase in the frequency of asymmetric label segregation by chance. Many studies have failed to control for the number of cell divisions after label delivery, perhaps due to the need for in-depth knowledge of cell cycle times. Although accurate cell cycle times have been established *in vitro* using live video microscopy, they will remain problematic for *in vivo* studies where division times may remain rough estimates. A low number of chromosomes in the model organism compounds this problem [10,21]. Although many thymidine analog labeling studies utilize a mouse model, organisms with as few as six or eight chromosomes have been employed [21].

Some chromosomes may be distributed randomly, necessitating chromosomal resolution of DNA to verify asymmetric segregation [22]. Recently, the development of chromosome orientation fluorescent *in situ* hybridization (CO-FISH) has made this possible [23,24]. Originally developed to examine obligate chromosomal inversions associated with isochromosome formation, CO-FISH uses Hoescht dye and UV radiation in conjunction with exonuclease III to remove BrdU-labeled DNA. Strand-specific telomeric probes can then be used to visualize the distribution of chromatids in metaphase. Recently, Rocheteau et al. [10] used CO-FISH to show that all chromatids participate in asymmetric DNA distribution during muscle regeneration in a subpopulation of muscle satellite cells. Likewise, Falconer et al. [25] provided evidence for the immortal DNA strand theory in the colon, where cell pairs displayed a higher degree of asymmetric chromosome distribution in CO-FISH assays than lung fibroblast and embryonic stem cell controls.

In addition to the limitations of thymidine analog labeling, ill-defined stem cell populations may include a mix of stem and progenitor cells, resulting in a failure to identify the appropriate subset of cells undergoing non-random chromosome distribution. Even in the hematopoietic compartment, where stem cells are defined by numerous surface antigens, additional characterization has led to new evidence in favor of asymmetric chromatid segregation [7,8]. Although previous studies failed to detect retention of a BrdU label in a murine hematopoietic stem cell (HSC) population, Wilson et al. recently demonstrated long term label retention in a subpopulation of these cells by including negative selection for CD34. These CD34^−^ HSCs outperformed CD34^+^ cells in serial transplantation and competitive reconstitution assays.

Studies have often failed to provide direct evidence that parent and daughter cells adopt distinct fates following asymmetric DNA segregation. Drosophila germ line stem cells (GSCs) and their progeny can be identified by established markers and their unique anatomical position. As GSCs undergo mitosis, one daughter remains attached to a fragment of somatic tissue called the hub, maintaining its identity as a stem cell, while its sister differentiates and migrates outward [26]. Exploiting this knowledge, Yadlapalli et al. [11] recently used thymidine analog labeling to demonstrate that GSCs undergoing divisions known to produce daughter cells with distinct fates do not segregate DNA asymmetrically. In contrast, Rocheteau et al. [10] employed DNA labeling techniques and fluorescent activated cell sorting to demonstrate that label retaining cells in regenerating muscle adopt a stem cell fate, while cells that receive newly synthesized DNA upregulate genes involved in myogenic differentiation.

Whether stem cells distribute their DNA asymmetrically will most likely depend on the cell population, tissue type, and the period of development under examination. It seems unlikely that chromatid segregation will follow a universal pattern. However, recent developments have exposed the limitations of current techniques and highlighted the need for appropriate experimental design. It may be premature to draw conclusions about the asymmetric segregation of DNA for even the most well characterized stem cell systems without identification of an appropriate subpopulation through the use of suitable markers and functional tests.

Based on the limitations of thymidine analog labeling outlined above, and data generated from previous studies, we suggest that future analysis of somatic stem cell chromosome segregation should strive to meet the following criteria:

1. The label must be shown to be present throughout the course of the chase.
2. The label must be incorporated into the “immortal” template DNA strand.
3. Characterization of label retaining stem cells must be rigorously established through the use of appropriate markers or functional tests.
4. The fate of both parent and daughter cells must be determined after asymmetric division.
5. The cell cycle time must be determined *in vivo* or *in vitro* and growth should not exceed two or three divisions.
6. Labeled cells should be shown to continually divide during the chase period.
7. The toxicity of the label should not affect the cell cycle.
8. Both growth and injury models should be utilized when possible.

The evidence in support or opposition of asymmetric DNA segregation is mixed. However, the biological advantage this distribution may impart to the parent cell remains uncertain. Retention of DNA could prevent the accumulation of mutations as initially suggested, but it may also play a critical role in maintaining stem cell quiescence and identity through epigenetic memory [27]. Future studies may require evaluation of DNA methylation patterns following asymmetric chromatid distribution.
